# A Resource Efficient System for On-Smartwatch Audio Processing

**DOI:** 10.1145/3636534.3698866

**Published:** 2024-12-04

**Authors:** Md Sabbir Ahmed, Arafat Rahman, Zhiyuan Wang, Mark Rucker, Laura E. Barnes

**Affiliations:** Department of Systems and Information Engineering University of Virginia, VA, USA

**Keywords:** Smartwatch, On-Device Audio Processing, Machine Learning, Wearable Computing

## Abstract

While audio data shows promise in addressing various health challenges, there is a lack of research on on-device audio processing for smartwatches. Privacy concerns make storing raw audio and performing post-hoc analysis undesirable for many users. Additionally, current on-device audio processing systems for smartwatches are limited in their feature extraction capabilities, restricting their potential for understanding user behavior and health. We developed a real-time system for on-device audio processing on smartwatches, which takes an average of 1.78 minutes (SD = 0.07 min) to extract 22 spectral and rhythmic features from a 1-minute audio sample, using a small window size of 25 milliseconds. Using these extracted audio features on a public dataset, we developed and incorporated models into a watch to classify foreground and background speech in real-time. Our Random Forest-based model classifies speech with a balanced accuracy of 80.3%.

## 1 Introduction

Advanced sensing technology, such as acoustic sensing available on smartwatches, has emerged as a promising method to detect health contexts such as social interactions [18]. However, the majority of the smartwatch-based systems explored physiological and non-acoustic biomarkers [2, 8].

Recently, audio processing has received much attention in real-time applications for problems such as pulmonary patient [3], breathing phase [22], breathing exercises [17], cough [11] and COVID-19 [6] detection. To collect audio data in naturalistic settings, researchers use earbuds [15, 22], smartwatches [3–5, 16], smartphones [6, 11], web-based system [6], and wearable headphones [10].

However, there is a dearth of research (e.g., [5]) that collects audio in a privacy-preserving way featurizing audio data on-board that smartwatch. Applications that store and process the raw audio later are invasive and may deter individuals from participating in studies [9] whereas quantification of the audio data may be acceptable [20]. There is little research [5] demonstrating holistic on-board feature extraction. Thus, existing systems may not fully realize the potential for in-depth understanding of individual behaviors and contexts.

Smartwatches, with their limited resources (e.g., low RAM and small battery size), present significant challenges for on-board audio processing. When extracting audio features using small windows (e.g., the widely adopted 25-millisecond window [13]), OutOfMemory errors frequently occur. For example, based on our empirical test, we found extracting decibel values by converting the audio power spectrum over 17 minutes can generate around 1 million values, and attempting to store such a large volume of data often results in memory overflow. Another challenge is the incompatibility of frameworks. While Python’s PyPi repository [1] offers libraries compatible with Windows, macOS, and Linux, many, such as Pydub [19], lack Wear OS-compatible versions. Additionally, modern computers support multiprocessing, which simplifies the use of libraries that rely on it. However, Wear OS faces limitations, such as not supporting POSIX semaphores, making multiprocessing more difficult.

Our platform makes the following contributions to the field of wearable computing:

- In addition to addressing issues such as framework incompatibility and OutOfMemory errors, we implemented optimizations (e.g., compressing feature values after encoding) to enable efficient on-device audio processing, reducing resource consumption. Our system can extract 22 spectral and rhythmic features in real-time, which, to our knowledge, exceeds the capabilities of existing on-smartwatch audio processing systems.
- Compared to the neural network-based model developed by [12] for classifying foreground and background speech, our Random Forest (RF) model achieves a similar balanced accuracy of 80.3% (vs. 80.4% for the existing model) but can be more lightweight and resource-efficient. This can make it particularly well-suited for running on low-resource devices.

## 2 System Development

### 2.1 On-Smartwatch Feature Extraction

To extract spectral and rhythmic features on the smartwatch, we customized Librosa [14] to ensure compatibility with Wear OS. Additionally, we streamlined PyAudioAnalysis [7], reducing its reliance on external libraries. We also updated the pipeline to ensure consistency across libraries in reading audio data and converting it to numerical values for feature extraction. The system rounds feature values to four decimal places, converts them to strings separating each feature value by comma, presents repeatedly same values by appending number of repetition and the value to a string “repeat_”, encodes, compresses, and stores the features in an npz file to minimize resource consumption. In total, the system extracts 22 audio features, including chromagram, RMS, 0th, 1st, and 2nd order polynomial coefficients, roll-off frequency, zero-crossing rate, energy, energy entropy, spectral bandwidth, contrast, flatness, centroid, spread, entropy, flux, melspectrogram, MFCCs, tempo, tempogram, Fourier tempogram, and tempogram ratio [7, 14], using a window size of 25 milliseconds and an overlap of 12.5 milliseconds. Feature files can be uploaded to Amazon S3 storage by pressing a button or when the watch is charging.

### 2.2 System Evaluation

**Figure 1:**
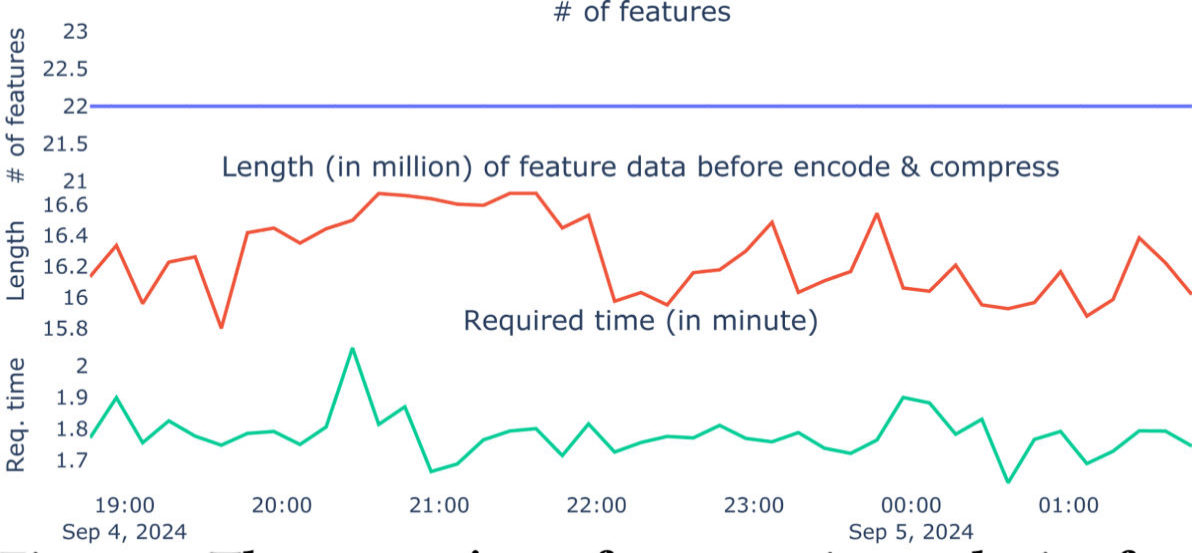
The system’s performance in on-device feature extraction.

We installed the application on two models of the Samsung Galaxy Watch, yielding consistent results. Our findings (Fig. 1) are based on data collected over a 7-hour period using a Galaxy Watch 5 Pro. The system collected and extracted features from 1-minute audio segments at 10-minute intervals. At the start of each probe, the system extracted 22 features. Before encoding and compressing, the length of extracted feature data ranged from 15.80 to 16.67 million (M) (mean = 16.26 M, SD = 0.26 M), and feature extraction took between 1.62 to 2.06 minutes (mean = 1.78 m, SD = 0.7 m).

### 2.3 Foreground Speech Detection

#### 2.3.1 Dataset:

Foreground speech refers to speech produced by the device wearer, while all other audio is considered background speech [12]. To develop foreground speech detection (FSD) models, we used a public dataset [12] comprising 113,820 speech instances, of which 23.71% are foreground and 76.29% are background, with each instance lasting 1 second.

#### 2.3.2 Model development:

After feature extraction (Section 2.1), we computed the minimum, maximum, mean, standard deviation, interquartile range (IQR), skewness, and kurtosis for each feature. For feature selection, we applied the Information Gain (IG) algorithm, and for machine learning (ML) model development, we employed several algorithms: KNN, SVC, Decision Tree (DT), Random Forest (RF), AdaBoost (AB), Gradient Boost (GB), and Extra Tree (ET). Hyper-parameter tuning was performed using the Bayesian search algorithm. To develop and validate the models, we utilized a nested cross-validation (CV) approach, which provides an unbiased performance estimate [21]. In nested CV, we applied a 10-fold CV in both the inner and outer loops, with the inner loop used for hyper-parameter tuning using training data and the outer loop for evaluation on test data.

#### 2.3.3 Performance:

**Figure 2:**
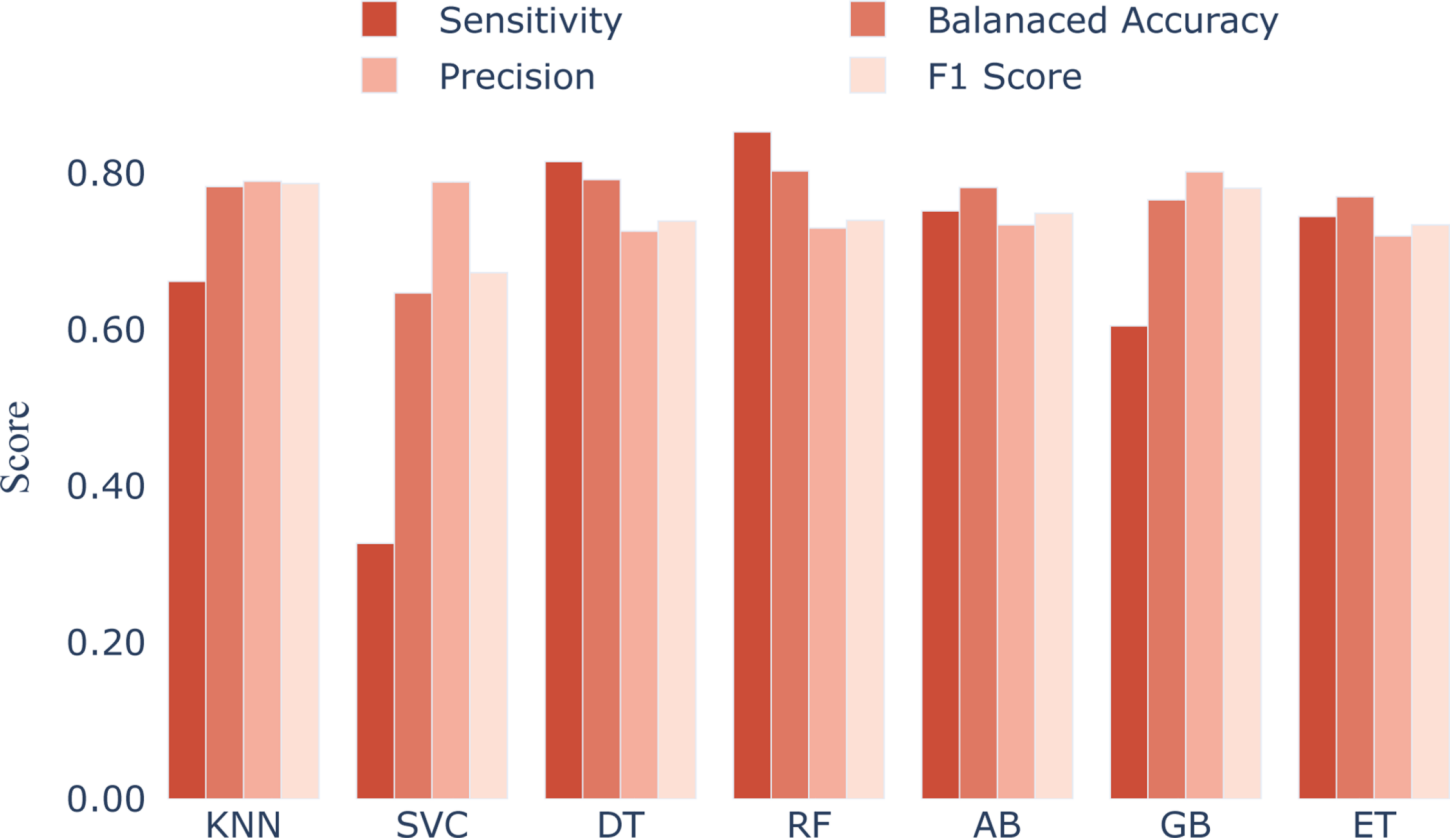
Performance of different ML models in classifying the foreground and background speeches.

Using IG selected 5 to 10 features, we explored how different ML models perform. Here, we presented the findings (Fig. 2) based on 9 important features which resulted in higher balanced accuracy. Among all ML models, RF had a higher balanced accuracy of 80.3% (Fig. 2). The sensitivity, macro F1, macro precision of the model were 85.3%, 74%, and 73% respectively.

## 3 Demonstration

**Figure 3:**
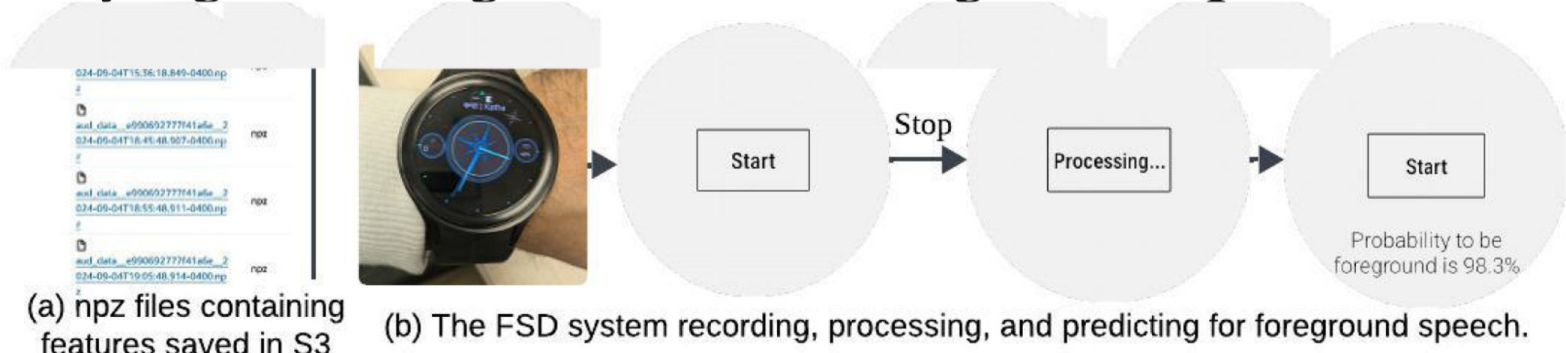
A watch user’s audio features in npz files in Amazon S3 (a) and the FSD system while recording, and predicting (b).

We will demonstrate^1^ the system’s (Fig. 3) ability to extract features on-board the smartwatch and save to the cloud. Additionally, we will showcase the real-time FSD classifier running on the watch. Attendees will have the opportunity to wear the watch and interact with the system using the user interface. We will also demonstrate how the feature files, uploaded to the cloud in a compressed and encrypted format, can be processed by executing a script to display their numerical values.

## CCS Concepts

- Human-centered computing → Ubiquitous and mobile computing systems and tools.

## Footnotes

1 Demo videos: https://github.com/BarnesLab/ACM-MobiCom-24-Demo
